# Urine-Based Biomarker Test Uromonitor^®^ in the Detection and Disease Monitoring of Non-Muscle-Invasive Bladder Cancer—A Systematic Review and Meta-Analysis of Diagnostic Test Performance

**DOI:** 10.3390/cancers16040753

**Published:** 2024-02-11

**Authors:** Anton P. Kravchuk, Ingmar Wolff, Christian Gilfrich, Ralph M. Wirtz, Paula Soares, Kay-Patrick Braun, Sabine D. Brookman-May, Lisa Kollitsch, Katharina Hauner, Martin Burchardt, Johannes Bründl, Maximilian Burger, Matthias May

**Affiliations:** 1 Department of Urology, St. Elisabeth Hospital Straubing, 94315 Straubing, Germany; 2 Department of Urology, University Medicine Greifswald, 17475 Greifswald, Germany; 3 STRATIFYER Molecular Pathology GmbH, 50935 Cologne, Germany; 4 IPATIMUP-Institute of Molecular Pathology and Immunology, University of Porto, 4200-135 Porto, Portugal; 5 Department of Pathology and Oncology, Faculty of Medicine, University of Porto, 4200-139 Porto, Portugal; 6 Institute of General Practice, Otto-von-Guericke-University Magdeburg, 39120 Magdeburg, Germany; 7 Department of Urology, Ludwig-Maximilians-University, 81377 Munich, Germany; 8 Johnson and Johnson Innovative Medicine, Research & Development, Spring House, PA 19477, USA; 9 Department of Urology and Andrology, Klinik Donaustadt, A1220 Vienna, Austria; 10 Department of Urology, University Hospital MRI-TUM (München rechts der Isar), 81675 Munich, Germany; 11 Department of Urology, Caritas St. Josef Medical Centre, University of Regensburg, 93053 Regensburg, Germany

**Keywords:** bladder cancer, detection, surveillance, urine-based diagnostic tests, urinary cytology, test accuracy, TERT, FGFR3, KRAS

## Abstract

**Simple Summary:**

Better tests are needed to detect serious bladder cancer (BC), additionally avoiding unnecessary follow-up tests. This study looked at Uromonitor^®^, a urine-based test, which checks for specific changes in DNA related to BC. Previous tests were good but not perfect, and more information is needed to use this new one in daily routine. We gathered and analyzed data from four studies using Uromonitor^®^, involving nearly 1200 urine tests. Results showed that Uromonitor^®^ detects existing BC with an accuracy of over 90%. It rarely indicates BC in cases where BC is absent (reaching 97% accuracy). In comparison with urinary cytology, Uromonitor^®^ spotted BC better. If applied in a group of 1000 people including around 15% with active BC, this test could prevent about 825 unnecessary cystoscopies while missing around 30 BC cases. However, more studies are needed to finally confirm the performance of Uromonitor^®^.

**Abstract:**

Optimal urine-based diagnostic tests (UBDT) minimize unnecessary follow-up cystoscopies in patients with non-muscle-invasive bladder-cancer (NMIBC), while accurately detecting high-grade bladder-cancer without false-negative results. Such UBDTs have not been comprehensively described upon a broad, validated dataset, resulting in cautious guideline recommendations. Uromonitor^®^, a urine-based DNA-assay detecting hotspot alterations in TERT, FGFR3, and KRAS, shows promising initial results. However, a systematic review merging all available data is lacking. Studies investigating the diagnostic performance of Uromonitor^®^ in NMIBC until November 2023 were identified in PubMed, Embase, Web-of-Science, Cochrane, Scopus, and medRxiv databases. Within aggregated analyses, test performance and area under the curve/AUC were calculated. This project fully implemented the PRISMA statement. Four qualifying studies comprised a total of 1190 urinary tests (bladder-cancer prevalence: 14.9%). Based on comprehensive analyses, sensitivity, specificity, positive-predictive value/PPV, negative-predictive value/NPV, and test accuracy of Uromonitor^®^ were 80.2%, 96.9%, 82.1%, 96.6%, and 94.5%, respectively, with an AUC of 0.886 (95%-CI: 0.851–0.921). In a meta-analysis of two studies comparing test performance with urinary cytology, Uromonitor^®^ significantly outperformed urinary cytology in sensitivity, PPV, and test accuracy, while no significant differences were observed for specificity and NPV. This systematic review supports the use of Uromonitor^®^ considering its favorable diagnostic performance. In a cohort of 1000 patients with a bladder-cancer prevalence of ~15%, this UBDT would avert 825 unnecessary cystoscopies (true-negatives) while missing 30 bladder-cancer cases (false-negatives). Due to currently limited aggregated data from only four studies with heterogeneous quality, confirmatory studies are needed.

## 1. Introduction

Urethrocytoscopy represents the gold standard for diagnostic evaluation of primary suspicion of urothelial carcinoma of the bladder (UCB) and in the oncological follow-up of non-muscle-invasive tumor stages (NMIBC) [1]. Among urine-based diagnostic tests (UBDT), only urinary cytology is widely established in clinical practice, although it exhibits limited sensitivity in low-grade (LG) UCB and heavily relies on the expertise of the examiner [2]. Over the past two decades, several UBDTs have been developed in an effort to avoid unnecessary cystoscopies in the oncological follow-up of NMIBC patients without compromising patient safety [3,4,5,6,7,8,9,10,11,12,13,14,15,16,17]. Cystoscopic follow-up, in particular, is associated with significant discomfort for NMIBC patients and significantly contributes to the high long-term costs of this disease [18]. In essence, the newer UBDTs can be differentiated into protein-based (Nuclear-Matrix-Protein 22^®^, Bladder-Tumor-Antigen^®^, ADXBladder^®^, cytokeratins), cell-based (UroVysion^®^, CellDetect^®^) approaches, as well as RNA-assays (Cxbladder Monitor^®^, Xpert BC^®^, MicroRNA) and DNA-based (Bladder EpiCheck^®^, Uromonitor^®^ test) [4]. The poor quality and mostly non-validated study results, which also lack robust evidence of a sufficiently high negative predictive value (NPV) and high specificity, represent a major problem resulting in the fact that these tests are not considered in guideline recommendations, e.g., those of the EAU [1,4,5]. A high NPV helps to minimize the risk of missing tumor recurrences during follow-up, which could lead to a prognostic deterioration, especially in high-grade (HG) UCB. High specificity, on the other hand, is a prerequisite for significantly reducing unnecessary cystoscopies. In a two-year-old meta-analysis, Laukhtina et al. demonstrated that UBDTs based on DNA-assays (Bladder EpiCheck^®^ and Uromonitor^®^ test) outperformed other urinary molecular marker tests in avoiding unnecessary cystoscopies. In their cohort associated with a recurrence rate of 18% (detected by cystoscopy), patients underwent an additional UBDT at the time of the scheduled routine follow-up cystoscopy. Calculated for 1000 NMIBC patients, Bladder EpiCheck^®^ and Uromonitor^®^ tests could avoid 693 and 696 unnecessary cystoscopies, respectively (corresponding to the rate of true-negative test results) [5]. Among the other urinary tests included in this meta-analysis that are based on protein or RNA, the rate of avoidable unnecessary cystoscopies was significantly lower [5].

The Uromonitor^®^ test based on DNA-assays analyzes hotspot alterations in three different genes (Telomerase Reverse Transcriptase/TERT, Fibroblast Growth Factor Receptor 3/FGFR3, and Kirsten Rat Sarcoma Oncogene/KRAS) associated with NMIBC using real-time qPCR (quantitative polymerase chain reaction) [19]. Apart from the previously cited meta-analysis by Laukhtina et al. published in 2021, which only included two studies evaluating the Uromonitor^®^ test, there is currently no systematic review of this test using a rigorous aggregated analysis of available study data [5,19,20]. Alongside with a prospective study validating the Uromonitor^®^ test currently conducted by our group in Germany, the aim of this work was to provide a comprehensive overview of the recent data on the urine-based and non-invasive Uromonitor^®^ test based on a systematic review, including a meta-analysis.

## 2. Materials and Methods (Evidence Acquisition)

### 2.1. Protocol Development and Protocol Registration

Prior to commencing this systematic review and the associated meta-analysis, and after thorough protocol development, this study was registered in the PROSPERO database according to its guidelines (PROSPERO registration ID: CRD42023473248). The protocol served as a guide for the entire systematic review and contains detailed information on the PICO questions (Population, Intervention, Control, Outcomes), search strategies, and planned analysis methods. This protocol is publicly accessible, providing transparency about the planned course of the systematic review. The PRISMA (Preferred Reporting Items for Systematic reviews and Meta-Analyses) statement guidelines were fully implemented in the creation of this systematic review (Tables S1 and S2 in the Supplementary Materials [21].

### 2.2. Literature Search

The literature search was conducted in the PubMed, Embase, Web of Science, Cochrane, Scopus databases, and medRxiv (a preprint server for medical articles submitted prior to, or in parallel with, peer-reviewed journal submission) to identify original studies related to the diagnostic value of the Uromonitor^®^ test in urine-based diagnosis of NMIBC patients, published until November 2023. The following keywords were used in our search strategy: (Uromonitor) AND (bladder cancer OR bladder carcinoma OR urothelial cancer OR urothelial carcinoma). 

This systematic review aimed to equally prioritize the defined criteria of UBDTs as outcomes (sensitivity, specificity, positive predictive value (PPV), NPV, and accuracy). We assessed certainty of evidence for each key outcome using the Grading of Recommendations Assessment, Development and Evaluation (GRADE) approach and created a “Summary of Findings” Table S3 in the Supplementary Materials using the GRADEpro guideline development tool (incorporating a categorization of the certainty of evidence (CoE) into very low, low, moderate, and high levels on the following domains: risk of bias, imprecision, inconsistency, indirectness, and publication bias) [22].

### 2.3. Inclusion and Exclusion Criteria

Criteria for study inclusion and exclusion were defined by the authors prior to conducting the literature search. Only studies meeting the following criteria were included:

1. Analysis of the diagnostic performance of the Uromonitor^®^ test for initial diagnosis and/or recurrence monitoring (surveillance) of NMIBC patients.
2. Data provision on the sensitivity, specificity, PPV, NPV, and accuracy of the Uromonitor^®^ test for initial diagnosis and/or recurrence monitoring of NMIBC patients. If one or more of these test quality criteria were not listed in the publication of the study, they were calculated based on the data matrix presented along with the study.

Study eligibility for the comparison of diagnostic performance of the Uromonitor^®^ test with urinary cytology were defined in accordance with the PICO approach. The inclusion criteria were as follows: (P) focus on adults (>18 yrs. old) with a (presumptive) diagnosis of bladder cancer, (I) undergoing Uromonitor^®^ test, (C) in which urinary cytology was performed as a comparator, and (O) evaluating one or more of the predefined endpoints sensitivity, specificity, PPV, NPV, and accuracy in (S) retrospective or prospective comparative studies.

Any studies not using standardized reference methods (cystoscopy and/or histopathological assessment) of the Uromonitor^®^ test were excluded. Review articles, editorials, conference abstracts lacking complete study information (see Section 2.4), letters to the editors, case reports, and study protocols without publication of results were also excluded.

### 2.4. Data Extraction

Two independent reviewers (AK, MM) extracted the following data from the included articles: Author name, publication year, number of patients, tumor stage and grade, presence of carcinoma in situ (CIS), recurrence rates, as well as sensitivity, specificity, PPV, NPV, test accuracy, and the number of true-positive (TP), true-negative (TN), false-positive (FP), and false-negative (FN) results. Any discrepancy in data extraction was resolved through the integration of a third reviewer (CG) in the form of a moderated consensus or majority judgment.

### 2.5. Risk of Bias Assessment

The risk of bias inherent in the included studies was assessed using the risk of bias (RoB) tool with the revised QUality Assessment of Diagnostic Accuracy Studies-2 (QUADAS-2) [23,24]. This allowed for a critical assessment of the quality of included studies and their potential bias. Each bias domain and the overall risk of bias were judged as “low”, “high”, or “unclear” risk of bias. The presence of confounders was determined by consensus and review of the literature. The ROBINS-I and risk-of-bias assessment of each study were conducted independently by two authors (AK, MM).

The index test was defined in detecting NMIBC by the Uromonitor^®^ test, with cystoscopy findings and histopathological evaluation serving as the reference.

### 2.6. Statistical Analysis and Meta-Analysis

Data on sensitivity, specificity, PPV, NPV, and test accuracy of the Uromonitor^®^ test were aggregated from the identified studies and reported. Subgroup analyses were planned if the data quality of the studies allowed for it, regarding primary NMIBC vs. surveillance setting, binary gender, and binary division into LG vs. HG-UCB. A diagnostic odds ratio (DOR) was defined to assess the predictive quality of the Uromonitor^®^ test in detecting UCB (DOR-1) and excluding UCB (DOR-2) (DOR including 95% confidence intervals/CI). Additionally, a receiver operating characteristic (ROC) curve was calculated to assess the diagnostic quality of the Uromonitor^®^ test, with the area under the curve (AUC) representing the ratio of sensitivity to 1-specificity, accompanied by a 95% CI (the *p*-value relates to the difference between the achieved AUC value and the random classifier line at an AUC value of 0.5).

A meta-analysis was conducted based on this systematic review to compare the diagnostic performance of the Uromonitor^®^ test and urinary cytology. The binary endpoints of this meta-analysis included sensitivity, specificity, PPV, NPV, and test accuracy. The meta-analysis focused on calculating the log-odds ratio, using a random-effects model. Forest plots were used to analyze and summarize the pooled odds ratio (OR) with a 95% CI to describe the diagnostic performance of Uromonitor^®^ test in comparison with urinary cytology. Heterogeneity between the studies identified in this meta-analysis was calculated using the Higgins/Thompson I^2^ test, with an I^2^ value greater than 35% indicating significant heterogeneity. Cochran’s Q tests were also used for this purpose, with *p* < 0.05 indicating significant heterogeneity. The graphical representation of publication bias was performed using funnel plots for all five endpoints, where the pooled logarithmically transformed odds ratios of the identified studies were plotted against the standard error. In Galbraith diagrams, as a measure of heterogeneity among the studies, the Z-score (including its 95% CI) was plotted against the inverse standard error.

The reported *p*-values are two-sided, and the significance level was considered statistically significant for all tests at *p* < 0.05. Data analysis was conducted using SPSS V.29 (IBM Corp., Armonk, NY, USA).

### 2.7. Standard Procedure of the Uromonitor^®^ Test

The Uromonitor^®^ test is a custom-made full working procedure developed and optimized for the detection in a real-time PCR platform of oncogene hotspot mutations in bladder cancer tumor cells, exfoliated to urine, particularly TERT promoter (124 and 146), FGFR3 codons (248 and 249), and KRAS codons (12/13 and 61) alterations (hotspot mutations) (Figure S4 in the Supplementary Materials). After collection, the urine was filtered using a pretreated 0.80 µm nitrocellulose syringe filter (Whatman^®^ Filter-Z612545, Merck, Darmstadt, Germany) containing a house-made conservative storage buffer. Per patient, ≥10 mL of urine was collected. A minimum of two filters per patient was required to perform adequate testing, with a minimum amount of 5 mL being used per filter. After the filtration process, the filters could be stored at 4 °C for at least a month, before being shipped at room temperature to the laboratory (U-Monitor Lda, Porto, Portugal) for further testing. High-molecular-weight DNA was extracted from the filters using the Norgen^®^ Plasma/Serum Cell-Free Circulating DNA Purification Mini Kit (Norgen Biotek Corp, Thorold, ON, Canada). TERT, FGFR3, and KRAS testing was performed on 25–50 ng of the extracted DNA. The extracted DNA was amplified and detected on a qPCR real-time machine (Applied Biosystems QS5, Thermo Fisher Scientific, Waltham, MA, USA) using the proprietary chemistry for amplification and detection, as provided in the Uromonitor^®^ test kit. Amplification signals were analyzed as recommended by the manufacturer (U-Monitor Lda, Porto, Portugal). If at least one of the screened alterations provided a positive result, then the test was positive.

## 3. Evidence Synthesis

### 3.1. Study Selection and Characterization of the Study Group

**Figure 1:**
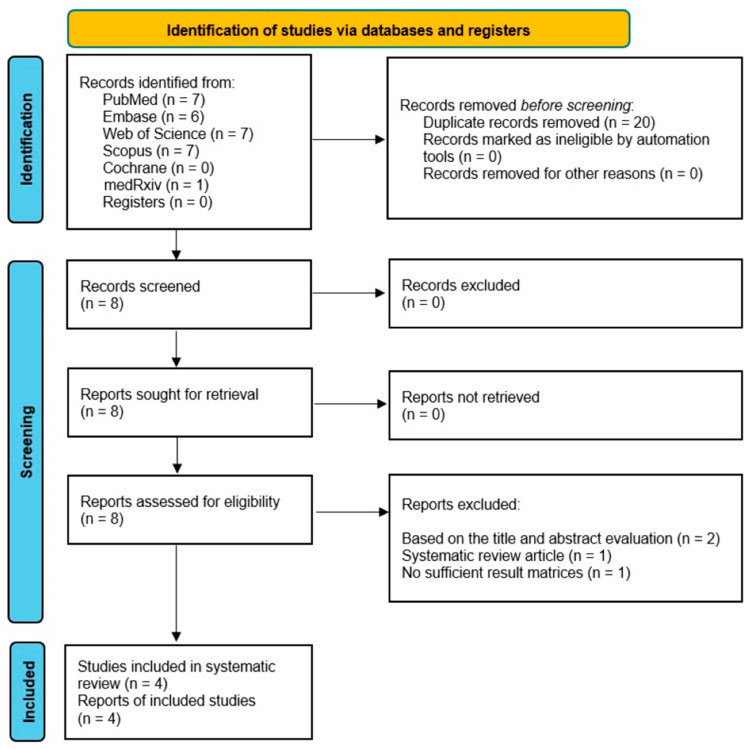
Preferred Reporting Items for Systematic reviews and Meta-Analysis flow of study selection.

The literature search identified eight articles (see flowchart in Figure 1). Four of the eight studies were excluded based on the title and abstract evaluation: one systematic review article, two incoherent original studies, and an EAU abstract with insufficient result matrices. Thus, four articles were included in the qualitative and, ultimately, in the quantitative synthesis (summarizing data based on 1190 nonduplicate Uromonitor^®^ tests) [19,20,25,26]. All four publications evaluated the diagnostic performance of the Uromonitor^®^ test in detecting NMIBC, with two of the articles also providing a comparative analysis of the performance of the Uromonitor^®^ test and urinary cytology [19,20].

A total of 1190 Uromonitor^®^ tests were conducted, of which 173 (14.5%) were positive. For the 177 tests (14.9%) in patients with histologically confirmed UCB, Table 1 provides an overview of the characterization of this group [19,20,25,26]. Out of 177 patients, 28 (16%) had primary UCB, while 149 had tumor recurrence (84%). Only two of the four studies provided complete information on tumor stages and patient gender (Table 1). Therefore, subgroup analyses were omitted in the subsequent calculations.

Due to the nature of the four studies included in the systematic review representing cohort (observational) studies, the CoE for all outcomes related to the five endpoints can only be rated as “low” (Table S3 in the Supplementary Materials).

Assessing the risk of bias in the four studies was documented in Figure S3 in the Supplementary Materials but was partly complicated by imprecise descriptions in the methodology sections of the four articles. For example, it was not specified in any of the four studies whether the urologist and the pathologist were blinded to the results of the Uromonitor^®^ test and urinary cytology during cystoscopy and histopathological assessments. Additionally, the studies did not indicate to what extent further investigations of FP test results were carried out (upper urinary tract examinations, patient follow-up, etc.).

### 3.2. Performance and Diagnostic Test Quality Criteria of the Uromonitor^®^ Test

**Figure 2:**
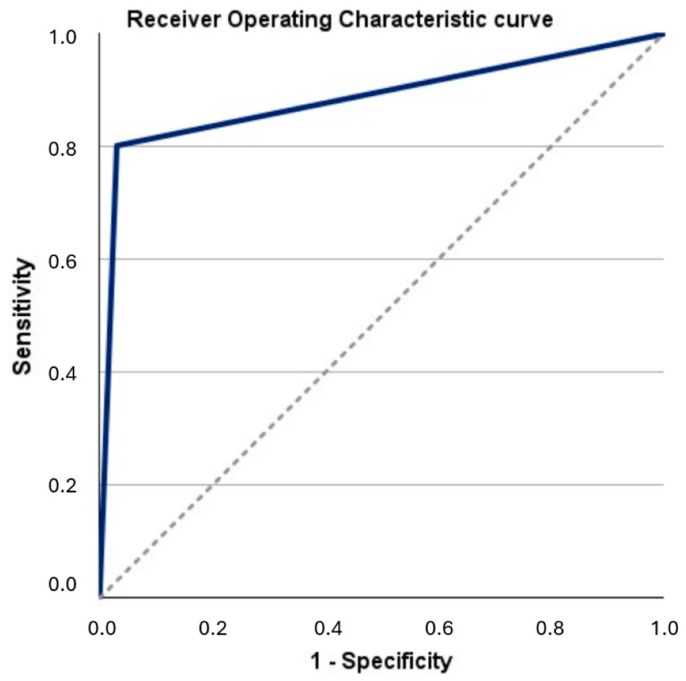
Receiver Operating Characteristic (ROC) curve for evaluating the diagnostic performance of the Uromonitor^®^ test. The ROC curve (solid line) depicts the trade-offs between sensitivity (vertical axis) and specificity (horizontal axis) using an aggregated study cohort comprising a total of 1190 tests. Each point on the ROC curve represents the performance of the Uromonitor^®^ test at different decision classification thresholds (cut-offs). The dashed line corresponds to the random boundary (area under curve/AUC of 0.5 = random classifier), while an ideal test would reach the upper-left corner (AUC = 1.0). The calculated AUC value serves as a quantitative measure of the overall performance of the Uromonitor^®^ test and is 0.89 with a 95% confidence interval of 0.85–0.92; *p* < 0.001.

The results of the four individual studies and the aggregated analyses on sensitivity, specificity, PPV, NPV, and test accuracy are presented in Table 2 [19,20,25,26]. Based on the aggregated analyses, the corresponding findings were 80.2%, 96.9%, 82.1%, 96.6%, and 94.5%. ROC analysis revealed an AUC of 0.886 (95% CI: 0.851–0.921; *p* < 0.001; Figure 2). With a positive Uromonitor^®^ test, the probability of actually detecting UCB increased by over 120 times (DOR-1: 128.5; 95% CI: 76.8–215; *p* < 0.001) compared to a negative test. Conversely, if the test result was negative, the probability of detecting UCB was reduced by 99.2% compared to a positive test result (DOR-2: 0.008; 95% CI: 0.005–0.013; *p* < 0.001).

### 3.3. Meta-Analysis of Diagnostic Performance Comparing Uromonitor^®^ Test and Urinary Cytology

**Figure 3:**
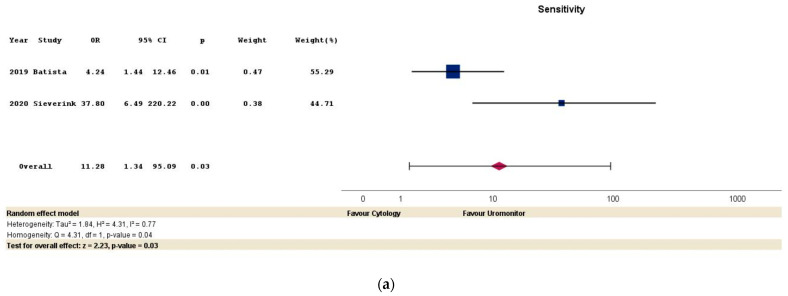

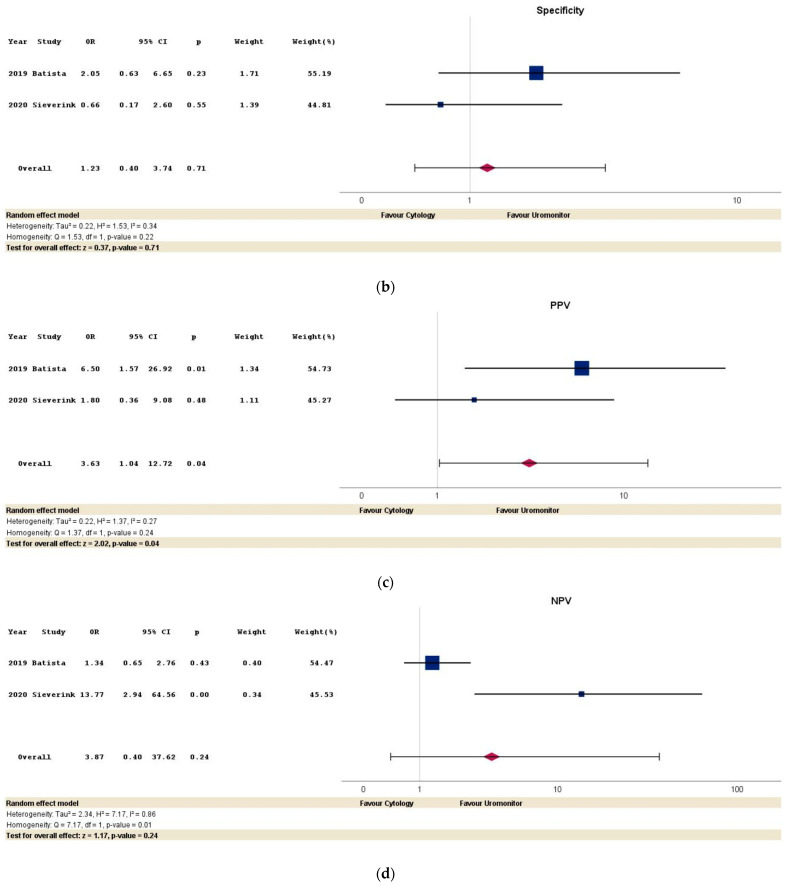

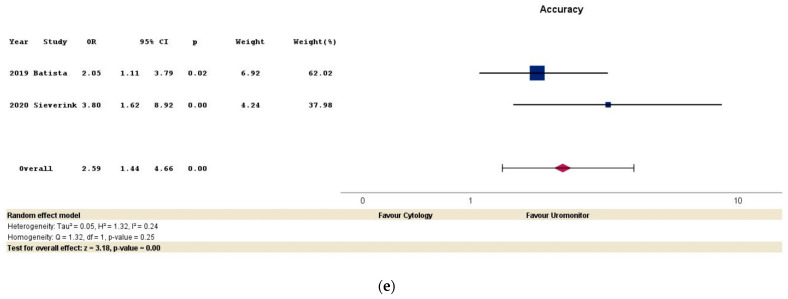
Forest plots for the meta-analysis of diagnostic performance of the Uromonitor^®^ test and urinary cytology in non-muscle-invasive bladder urothelial carcinoma. Each forest plot represents one of the following endpoints: sensitivity (**a**), specificity (**b**), positive predictive value (**c**), negative predictive value (**d**), and test accuracy (**e**). The pooled effect sizes and 95% confidence intervals for each endpoint are depicted for individual studies. The size of the bars corresponds to the weight of each study in the meta-analysis. Cochran’s Q and I^2^ statistic are used to assess heterogeneity among the studies. Data were extracted based on a systematic literature review and meta-analysis from two comparative studies comprising a total of 282 Uromonitor^®^ tests and 139 urinary cytology tests [19,20]. Legend: NPV, negative predictive value; OR, odds ratio; PPV, positive predictive value.

Forest plots for sensitivity, specificity, PPV, NPV, and test accuracy in the two studies comparing the diagnostic performance of the Uromonitor^®^ test and urinary cytology are shown in Figure 3a–e [19,20]. Based on the aggregated analyses of both studies, significant advantages were found in favor of the Uromonitor^®^ test in terms of sensitivity (OR: 11.28; 95% CI: 1.34–95.09; *p* = 0.03), PPV (OR: 3.63; 95% CI: 1.04–12.72; *p* = 0.04), and test accuracy (OR: 2.59; 95% CI: 1.44–4.66; *p* < 0.001). However, no significant differences were observed in specificity (OR: 1.23; 95% CI: 0.40–3.74; *p* = 0.71) and NPV (OR: 3.87; 95% CI: 0.40–37.62; *p* = 0.24).

Significant heterogeneity was present for sensitivity and NPV among the two included studies in the forest plots, as determined by Cochran’s Q test and I^2^ test (for these criteria, I^2^ values of 77% and 86%, respectively, are estimated). In contrast, the forest plots for specificity, PPV, and test accuracy showed negligible heterogeneity (Figure 3a–e). The graphical representation of publication bias was completed using funnel plots (Figure S1a–e in the Supplementary Materials), while the general heterogeneity of the estimated effect size was graphically presented using Galbraith plots (Figure S2a–e in the Supplementary Materials).

## 4. Discussion

### 4.1. Discussion of Results in the Scope of Current Literature

UBDTs are increasingly used in clinical practice for the initial assessment of UCB and, in particular, as a part of follow-up evaluations in patients with NMIBC [1,2,3,4,5,27]. Among these tests, urinary cytology is the most established and has been standardized for over 75 years [1,2,3,4,5,27,28,29]. Its sensitivity is high for HG disease, but insufficient to detect LG-UCB [1,2,3,4,5,26]. Moreover, interpreting urinary cytology results can be challenging, especially when urine conditions change (e.g., urinary tract infections, bladder stones, topical instillation therapy), and a substantial interobserver variability has been noted [30,31]. Due to these limitations, numerous other UBDTs have been developed to improve sensitivity, especially for LG-UCB, although at the expense of lower specificity when compared to urinary cytology [1,2,3,4,5]. Despite some of the new UBDTs showing excellent diagnostic performance and test accuracy in studies, their widespread adoption in clinical practice remains limited [1,2,3,4,5]. High reproducibility of results is a key prerequisite for their broad use, a criterion that only a few of the new UBDTs have met thus far [1]. The current European Association of Urology (EAU) guidelines suggest that four commercially available UBDTs may have the highest potential: ADX-Bladder^®^ (protein-based), Cx-Bladder Monitor^®^ (RNA-based), Xpert BC^®^ (RNA-based), and EpiCheck^®^ (DNA-based) [1]. In 2019, the first results of the DNA-based Uromonitor^®^ test in NMIBC patients were published [20]. This biomarker test assesses three hotspot alterations: TERT mutations detected in up to 80% of NMIBC cases, FGFR-3 mutations in up to 70%, and mutations of RAS oncogenes in approximately 11–13% [25,26]. The potential of the Uromonitor^®^ test for NMIBC patients is enormous. However, its various indications for optional use still require a more robust and higher level of evidence (Table 3) [19,20,25,26,27,28,29,30,31,32,33,34].

The most secure data are available for the diagnostic performance of the Uromonitor^®^ test, particularly in the surveillance of NMIBC patients [19,20,25,26]. A high rate of true-negative test results assists colleagues in uro-oncological follow-up in avoiding unnecessary cystoscopies [35,36,37,38]. This significantly contributes to the patients’ quality of life, prevents cystoscopy-associated side effects (urgency, urinary tract infections, urethral injuries), and reduces treatment costs [4,5,18]. In their systematic review from 2021, Laukhtina et al. analyzed that the UBDTs highlighted in the EAU guidelines—ADX-Bladder^®^ (protein-based), Cx-Bladder Monitor^®^ (RNA-based), Xpert BC^®^ (RNA-based), and EpiCheck^®^ (DNA-based)—could avoid 501, 485, 638, and 693 unnecessary cystoscopies, respectively (out of 1000 scheduled cystoscopies within a cohort associated with an NMIBC rate of 18%) [5].

As visualized using a Receiver Operating Characteristic (ROC) curve (Figure 2), the Uromonitor^®^ test achieved an AUC of 0.89 (95% confidence interval of 0.85–0.92) in a pooled patient cohort, associated with a UCB prevalence of approximately 15%. This indicates a satisfactory balance between sensitivity and specificity under these conditions.

**Figure 4:**
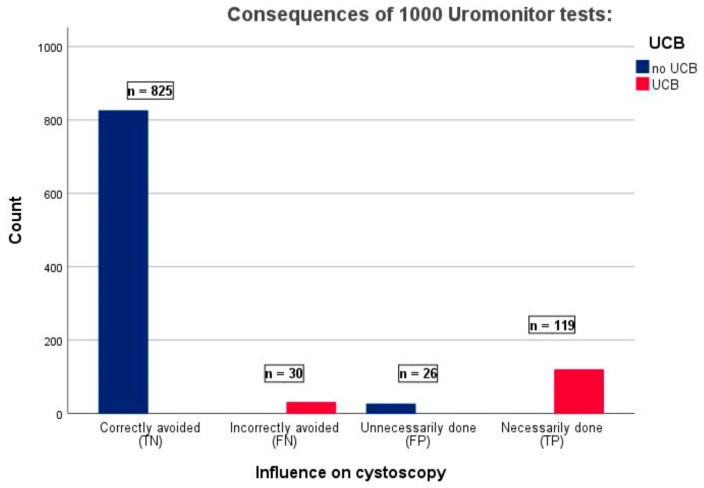
Net benefit and avoided cystoscopic assessments extrapolated to 1000 urinary tests in a cohort with 149 (14.9%) UCBs, where the Uromonitor^®^ provided a total of 145 positive (FP + RP) and 855 negative (FN + RN) test results. Legend: FN, false-negative urinary test result; FP, false-positive urinary test result; TN, true-negative urinary test result; TP, true-positive urinary test result; UCB, urothelial carcinoma of the bladder.

In this analysis, the Uromonitor^®^ test achieved the highest number of true-negative test results (696 cases) [5]. Extrapolating our aggregated analysis data to 1000 scheduled cystoscopies with a 14.9% NMIBC detection rate, the Uromonitor^®^ test could potentially avoid 825 unnecessary cystoscopies (representing TN test results; Figure 4). To provide a clearer comparison with the results of the systematic review by Laukhtina et al., we developed a new proxy for the diagnostic performance of UBDTs in NMIBC surveillance: ((TN rate + FN rate)/FN rate). Applying this formula to the Uromonitor^®^ tests results in a quotient of 855/30 = 28.5, which translates into a prevention of 28.5 unnecessary cystoscopies at the cost of missing one NMIBC recurrence (Figure 4). Applying this “Avoid-Proxy” to the data from Laukhtina et al.’s systematic review, the recommended UBDTs had the following “Avoid-Proxies”: ADX-Bladder^®^ 7.4, Cx-Bladder Monitor^®^ 33.3, Xpert BC^®^ 13.5, EpiCheck^®^ 15.7, and Uromonitor^®^ 70.6 [5]. Batista et al. reported an unusually high false-negative rate for the Uromonitor^®^ test as opposed to the other three studies [19,20,25,26]. Based on their results, an “Avoid-Proxy” of 6.1 and 4.8 was calculated for the Uromonitor^®^ test and urine cytology, respectively. In contrast, a significant difference in favor of the Uromonitor^®^ test (compared to urine cytology) is revealed based on results shown by Sieverink et al.: 30.5 vs. 3.1 [19].

Although the “Avoid-Proxy” depends on the proportion of NMIBC patients in a pertinent cohort, we believe that it provides a useful measure in comparative studies of the diagnostic performance of multiple UBDTs. Similarly, it is meaningful (a kind of “Avoid-Proxy vice versa”) to consider the percentage of patients with negative test results in whom an NMIBC recurrence was missed (FN × 100%/(TN rate + FN rate) or 100%/“Avoid-Proxy”). According to the data from Laukhtina et al., the UBDTs supported in the EAU guidelines had the following values: ADX-Bladder^®^ 13.5%, Cx-Bladder Monitor^®^ 3%, Xpert BC^®^ 7.4%, and EpiCheck^®^ 6.4% [5]. The Uromonitor^®^ test achieved corresponding figures of 1.4% and 3.5% in Laukhtina et al.’s systematic review and in our dataset, respectively.

### 4.2. Limitations and Perspectives

This study has several limitations that should be considered while interpreting the results. The most significant limitation is the continued insufficient aggregate case number of published Uromonitor^®^ tests (*n* = 1190 with a confirmed UCB rate of 14.9%) and the partially incomplete characterization of study groups within the identified four articles. This led us to decide against conducting subgroup analyses, which would have been essential for a test evaluation. Due to the design of the studies included in this analysis, representing cohort studies, only a “low” CoE could be assigned to the calculated results. Therefore, future prospective, well-designed comparative studies with clear patient characterizations are necessary. It is also important to note that the article by Ramos et al. is currently undergoing a peer-review process and was identified by us on the preprint server medRxiv [26]. None of the studies we identified was structured to assess the influence of topical instillation therapy or other interfering factors on test performance. It was also not clear from the studies how false-positive test results were followed up. Another limitation is the lack of a consistent assignment of the specific histology to the 35 FN test results, rendering it difficult to determine whether HG findings were missed by UBDT. Moreover, the diagnostic performance could not be reflected on basis of tissue-based subtyping (molecular analysis) and it is tempting to speculate whether the false negative results of the Uromonitor^®^ test are observed in non-luminal, less differentiated tumors with lower mutational frequencies of FGFR3 and KRAS particularly. In addition, it was not apparent from the methodological design of the studies whether there was blinding of the urinary test results to the urologist performing cystoscopy and potentially the pathologist examining the tissue. Furthermore, none of the four studies showed whether the Uromonitor^®^ test exhibits interobserver and/or intraobserver variability or whether different urine samples from the same patient and time always correlated with the same test result. The meta-analysis comparing the diagnostic performance of the Uromonitor^®^ test with urinary cytology is based solely on data from two studies comprising 282 and 139 urinary tests, respectively. To address the heterogeneity between the two studies regarding sensitivity and NPV endpoints (with I^2^ of 0.77 and 0.86, respectively), all meta-analyses were strictly conducted using random-effect models. Of note, to assess the heterogeneity of the studies included and the risk of publication bias, results of the Funnel plots must be interpreted in conjunction with other methods for assessing bias, e.g., statistical tests for asymmetry like the Egger test and Galbraith diagrams included in the Supplementary Materials. However, considering the rather high heterogeneity of studies, especially in terms of sensitivity and NPV of the Uromonitor^®^ test, results need to be interpreted with caution. Therefore, again, prospective-randomized trials seem warranted as mentioned above.

Another limitation is the fact that a study with 206 Uromonitor^®^ tests could not be included in the systematic review since the results were only available as an abstract from the 2023 Congress of the European Association of Urology, lacking the necessary study information [39]. Finally, in line with other UBDT studies, the studies considered for the systematic review only analyzed the diagnostic performance of the urinary test and did not examine the oncological and prognostic impact [3,40,41,42]. Thus, it is now time to develop prospective randomized studies that assess the impact of partially urine-based versus purely cystoscopy-oriented follow-up protocols on relevant oncological endpoints and on basis of matched tissue diagnostics.

## 5. Conclusions

The results of this systematic review support the use of the Uromonitor^®^ test due to its favorable diagnostic performance in patients with non-muscle-invasive bladder cancer, especially during oncological follow-up. Opposed to urinary cytology, this test exhibits significant advantages in terms of sensitivity, positive predictive value, and test accuracy. The “Avoid-Proxy” developed and presented in this article represents the ratio of test negativity to the proportion of false negative test results. For the Uromonitor^®^ test, an “Avoid-Proxy” of 28.5 was found, meaning that 28.5 unnecessary cystoscopies can be avoided at the cost of missing one recurrence. The time is ripe for studies with direct evidence that compare partially urine-based surveillance with purely cystoscopy-oriented follow-up protocols in terms of their impact on relevant oncological and patient-reported endpoints. Results of this systematic review provide the rationale for developing these studies using the Uromonitor^®^ test in patients with non-muscle-invasive bladder cancer.

## Figures and Tables

**Table 1:** Characterization of the 177 patients with (non-)muscle-invasive urothelial bladder carcinoma from the four recently published studies assessing the diagnostic performance of the Uromonitor^®^ test [19,20,25,26].

First Author, Year of Publication	UCB-Positive Cases/Total Tests (%)	Primary UCB/ Recurrent UCB	Tumor Stages	Sex
Batista, 2019 [20]	62/185 (33.5%)	28/34	CIS, *n* = 5; Ta, *n* = 32; T1, *n* = 12; T2, *n* = 2; n.a., *n* = 11	n.a.
Sieverink, 2020 [19]	29/97 (29.9%)	0/29	PUNLMP, *n* = 2; CIS, *n* = 7; Ta, *n* = 17; T1, *n* = 3	♀, *n* = 9; ♂, *n* = 20
Azawi, 2023 [25]	39/380 (10.3%)	0/39	n.a.	♀, *n* = 11; ♂, *n* = 28
Ramos, 2023 [26]	47/528 (8.9%)	0/47	Ta, *n* = 31; T1, *n* = 14; T2, *n* = 2	n.a.
Aggregated analysis	177/1190 (14.9%)	28 (16%)/ 149 (84%)	PUNLMP, *n* = 2 (1%); CIS, *n* = 12 (7%); Ta, *n* = 80 (45%); T1, *n* = 29 16%); T2, *n* = 4 (2%) n.a., *n* = 50 (28%)	♀, *n* = 20 (11%); ♂, *n* = 48 (27%); n.a., *n* = 109 (62%)

Legend: CIS, carcinoma in situ; n.a., not available; PUNLMP, papillary urothelial neoplasm of low malignant potential; UCB, urothelial bladder carcinoma; ♀, female; ♂, male.

**Table 2:** The performance of the Uromonitor^®^ test in urine-based diagnostics of non-muscle-invasive urothelial bladder carcinoma, based on the currently available four studies [19,20,25,26].

Criteria	Batistia [20]	Sieverink [19]	Azawi [25]	Ramos [26]	Aggregated Analysis
Tests conducted	185	97	380	528	1190
Proportion of UCB (%)	62 (33.5%)	29 (29.9%)	39 (10.3%)	47 (8.9%)	177 (14.9%)
Sensitivity	62.9%	93.1%	89.7%	87.2%	80.2%
	(39/62)	(27/29)	(35/39)	(41/47)	(142/177)
Specificity	95.1%	86.8%	96.2%	99.4%	96.9%
	(117/123)	(59/68)	(328/341)	(478/481)	(982/1013)
PPV	86.7%	75%	89.7%	93.2%	82.1%
	(39/45)	(27/36)	(35/48)	(41/44)	(142/173)
NPV	83.6%	96.7%	98.8%	98.8%	96.6%
	(117/140)	(59/61)	(328/332)	(478/484)	(982/1017)
Accuracy	84.3%	88.7%	95.5%	98.3%	94.5%
	(156/185)	(86/97)	(363/380)	(519/528)	(1124/1190)

Legend: NPV, negative predictive value; PPV, positive predictive value; UCB, urothelial bladder carcinoma.

**Table 3:** Theoretical potential of the Uromonitor^®^ test in the setting of NMIBC [19,20,25,26,32,33,34].

Option	Data Availability	Potential
Surveillance in NMIBC patients	pDa is based on a total of 1127 tests [19,20,25,26], showing a high negative predictive value in this systematic review.	♣♣♣
Screening in NMIBC risk groups or those with a predisposition to risk	No pDa available for this, but pDL for 63 tests in the primary setting [20], demonstrating a sensitivity of 50% and specificity of 100%.	♣♣
Exploration in cases of inconclusive findings from other UBDTs or cystoscopy	Superiority in terms of sensitivity, positive predictive value (PPV), and test accuracy compared to urinary cytology was demonstrated in this systematic review, with pDL based on only two studies [19,20].	♣♣
(Supportive) Indication for topical (intravesical) or systemic Erdafitinib therapy	No pDa available for this, but high concordance between urine-based and tissue-based detection of FGFR3 mutations was shown, including the work of the BRIDGister group [32,33].	♣
Response monitoring of topical (intravesical) Erdafitinib therapy	No pDa available; however, the effectiveness of topical Erdafitinib therapy for UBC patients needs to be demonstrated first [34].Change of FGFR3 status in recurrences after targeted treatment (potential evidence of clonality of NMIBC).	♣

Legend: FGFR3, fibroblast growth factor receptor 3; NMIBC, non-muscle-invasive bladder cancer; NPV, negative predictive value; pDa, published data in peer-reviewed journals; UBC, urothelial bladder carcinoma; UBDT, urine-based diagnostic tests; ♣, no medium-term feasibility; ♣♣, potential medium-term feasibility; ♣♣♣, potential short-term feasibility.

## Data Availability

All data generated or analyzed during this study are included in this article and its online Supplementary Materials. Further inquiries can be directed to the corresponding author.

## Supplementary Materials

The following supporting information can be downloaded at: https://www.mdpi.com/article/10.3390/cancers16040753/s1, Figure S1a–e: Funnel plots of the meta-analysis; Figure S2a–e: Galbraith plots of the meta-analysis; Figure S3: Risk of bias (RoB) assessment of the four studies included; Figure S4: Workflow of the Uromonitor^®^ test; Table S1: PRISMA 2020 main checklist; Table S2: PRISMA abstract checklist; Table S3: certainty of evidence (CoE) of the recommendation according to GRADE.
