# Supplementary material for: Urine-Based Biomarker Test Uromonitor® in the Detection and Disease Monitoring of Non-Muscle-Invasive Bladder Cancer—A Systematic Review and Meta-Analysis of Diagnostic Test Performance
Source: Cancers (Basel). 2024 Feb 11;16(4):753. doi: 10.3390/cancers16040753 (PMC10886463; doi:10.3390/cancers16040753)
Supplement: Supplementary file 1 [file cancers-16-00753-s001.zip › cancers-2815456-supplementary.pdf]

Tabular listing of results or elements included in Supplementary Materials

PRISMA 2020 Main Checklist (Table S1)	*pages 2–7*
PRISMA Abstract Checklist (Table S2)	*pages 8–9*
Funnel plots of the meta-analysis (Figure S1a-e)	*pages 10–12*
Galbraith plots of the meta-analysis (Figure S2a-e)	*pages 13–15*
Risk of Bias (RoB) Assessment of the four studies included (Figure S3)	*page 16–17*
Certainty of Evidence (CoE) of the recommendation according to GRADE (Table S3)	*pages 18–19*
Workflow of the Uromonitor® test (Figure S4)	*page 20–21*

Table S1: PRISMA 2020 Main Checklist

Topic	No.	Item	Location where item is reported
**TITLE**			
**Title**	1	Identify the report as a systematic review.	is integrated
**ABSTRACT**			
**Abstract**	2	See the PRISMA 2020 for Abstracts checklist	has taken place
**INTRODUCTION**			
**Rationale**	3	Describe the rationale for the review in the context of existing knowledge.	is integrated
**Objectives**	4	Provide an explicit statement of the objective(s) or question(s) the review addresses.	is integrated
**METHODS**			
**Eligibility criteria**	5	Specify the inclusion and exclusion criteria for the review and how studies were grouped for the syntheses.	is integrated
**Information sources**	6	Specify all databases, registers, websites, organizations, reference lists and other sources searched or consulted to identify studies. Specify the date when each source was last searched or consulted.	is integrated
**Search strategy**	7	Present the full search strategies for all databases, registers and websites, including any filters and limits used.	is integrated

Topic	No.	Item	Location where item is reported
**Selection process**	8	Specify the methods used to decide whether a study met the inclusion criteria of the review, including how many reviewers screened each record and each report retrieved, whether they worked independently, and if applicable, details of automation tools used in the process.	is integrated
**Data collection process**	9	Specify the methods used to collect data from reports, including how many reviewers collected data from each report, whether they worked independently, any processes for obtaining or confirming data from study investigators, and if applicable, details of automation tools used in the process.	is integrated
**Data items**	10a	List and define all outcomes for which data were sought. Specify whether all results that were compatible with each outcome domain in each study were sought (e.g., for all measures, time points, analyses), and if not, the methods used to decide which results to collect.	is integrated
	10b	List and define all other variables for which data were sought (e.g., participant and intervention characteristics, funding sources). Describe any assumptions made about any missing or unclear information.	is integrated
**Study risk of bias assessment**	11	Specify the methods used to assess risk of bias in the included studies, including details of the tool(s) used, how many reviewers assessed each study and whether they worked independently, and if applicable, details of automation tools used in the process.	is integrated

Topic	No.	Item	Location where item is reported
**Effect measures**	12	Specify for each outcome the effect measure(s) (e.g., risk ratio, mean difference) used in the synthesis or presentation of results.	is integrated
**Synthesis methods**	13a	Describe the processes used to decide which studies were eligible for each synthesis (e.g., tabulating the study intervention characteristics and comparing against the planned groups for each synthesis (item 5)).	is integrated
	13b	Describe any methods required to prepare the data for presentation or synthesis, such as handling of missing summary statistics, or data conversions.	is integrated
	13c	Describe any methods used to tabulate or visually display results of individual studies and syntheses.	is integrated
	13d	Describe any methods used to synthesize results and provide a rationale for the choice(s). If meta-analysis was performed, describe the model(s), method(s) to identify the presence and extent of statistical heterogeneity, and software package(s) used.	is integrated
	13e	Describe any methods used to explore possible causes of heterogeneity among study results (e.g., subgroup analysis, meta-regression).	is integrated
	13f	Describe any sensitivity analyses conducted to assess robustness of the synthesized results.	is integrated
**Reporting bias assessment**	14	Describe any methods used to assess risk of bias due to missing results in a synthesis (arising from reporting biases).	is integrated
**Certainty assessment**	15	Describe any methods used to assess certainty (or confidence) in the body of evidence for an outcome.	is integrated

Topic	No.	Item	Location where item is reported
RESULTS			
Study selection	16a	Describe the results of the search and selection process, from the number of records identified in the search to the number of studies included in the review, ideally using a flow diagram.	is integrated
	16b	Cite studies that might appear to meet the inclusion criteria, but which were excluded, and explain why they were excluded.	is integrated
Study characteristics	17	Cite each included study and present its characteristics.	is integrated
Risk of bias in studies	18	Present assessments of risk of bias for each included study.	is integrated
Results of individual studies	19	For all outcomes, present, for each study: (a) summary statistics for each group (where appropriate) and (b) an effect estimate(s) and its precision (e.g. confidence/credible interval), ideally using structured tables or plots.	is integrated
Results of syntheses	20a	For each synthesis, briefly summarize the characteristics and risk of bias among contributing studies.	is integrated
	20b	Present results of all statistical syntheses conducted. If meta-analysis was done, present for each the summary estimate and its precision (e.g., confidence/credible interval) and measures of statistical heterogeneity. If comparing groups, describe the direction of the effect.	is integrated
	20c	Present results of all investigations of possible causes of heterogeneity among study results.	is integrated

Topic	No.	Item	Location where item is reported
	20d	Present results of all sensitivity analyses conducted to assess the robustness of the synthesized results.	is integrated
Reporting biases	21	Present assessments of risk of bias due to missing results (arising from reporting biases) for each synthesis assessed.	is integrated
Certainty of evidence	22	Present assessments of certainty (or confidence) in the body of evidence for each outcome assessed.	is integrated
DISCUSSION			
Discussion	23a	Provide a general interpretation of the results in the context of other evidence.	is integrated
	23b	Discuss any limitations of the evidence included in the review.	is integrated
	23c	Discuss any limitations of the review processes used.	is integrated
	23d	Discuss implications of the results for practice, policy, and future research.	is integrated
OTHER INFORMATION			
Registration and protocol	24a	Provide registration information for the review, including register name and registration number, or state that the review was not registered.	is integrated
	24b	Indicate where the review protocol can be accessed, or state that a protocol was not prepared.	is integrated
	24c	Describe and explain any amendments to information provided at registration or in the protocol.	is integrated

Topic	No.	Item	Location where item is reported
**Support**	25	Describe sources of financial or non-financial support for the review, and the role of the funders or sponsors in the review.	The authors received no financial support for the research, authorship and/or publication of this article.
**Competing interests**	26	Declare any competing interests of review authors.	was integrated
**Availability of data, code and other materials**	27	Report which of the following are publicly available and where they can be found template data collection forms; data extracted from included studies; data used for all analyses; analytic code; any other materials used in the review.	upon request from the corresponding author

From references in the main manuscript [21]. For more information, visit: www.prisma-statement.org

Table S2: PRIMSA Abstract Checklist

Topic	No.	Item	Reported?
**TITLE**			
**Title**	1	Identify the report as a systematic review.	Yes
**BACKGROUND**			
**Objectives**	2	Provide an explicit statement of the main objective(s) or question(s) the review addresses.	Yes
**METHODS**			
**Eligibility criteria**	3	Specify the inclusion and exclusion criteria for the review.	Yes
**Information sources**	4	Specify the information sources (e.g. databases, registers) used to identify studies and the date when each was last searched.	Yes
**Risk of bias**	5	Specify the methods used to assess risk of bias in the included studies.	Yes
**Synthesis of results**	6	Specify the methods used to present and synthesize results.	Yes
**RESULTS**			
**Included studies**	7	Give the total number of included studies and participants and summarise relevant characteristics of studies.	Yes
**Synthesis of results**	8	Present results for main outcomes, preferably indicating the number of included studies and participants for each. If meta-analysis was done, report the summary estimate and confidence/credible interval. If comparing groups, indicate the direction of the effect (i.e. which group is favoured).	Yes
**DISCUSSION**			
**Limitations of evidence**	9	Provide a brief summary of the limitations of the evidence included in the review (e.g. study risk of bias, inconsistency and imprecision).	Yes

Topic	No.	Item	Reported?
**Interpretation**	10	Provide a general interpretation of the results and important implications.	Yes
OTHER			
**Funding**	11	Specify the primary source of funding for the review.	Yes
**Registration**	12	Provide the register name and registration number.	Yes

From references in the main manuscript [21].

Figure S1a-e: Funnel Plots

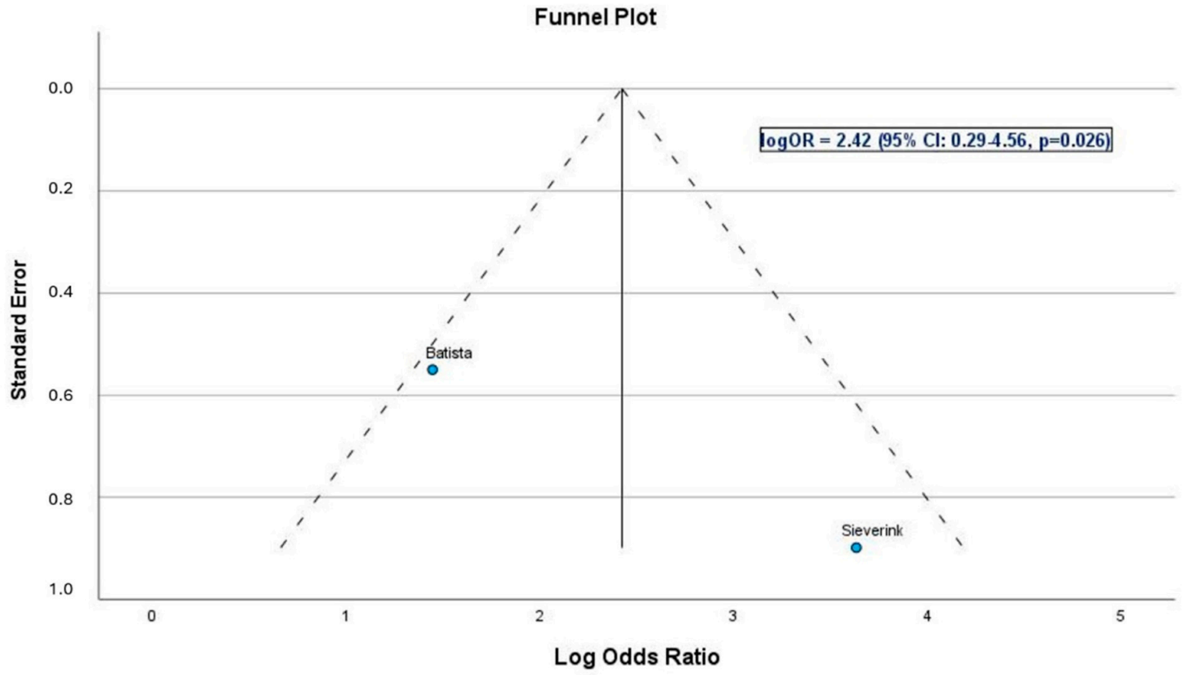

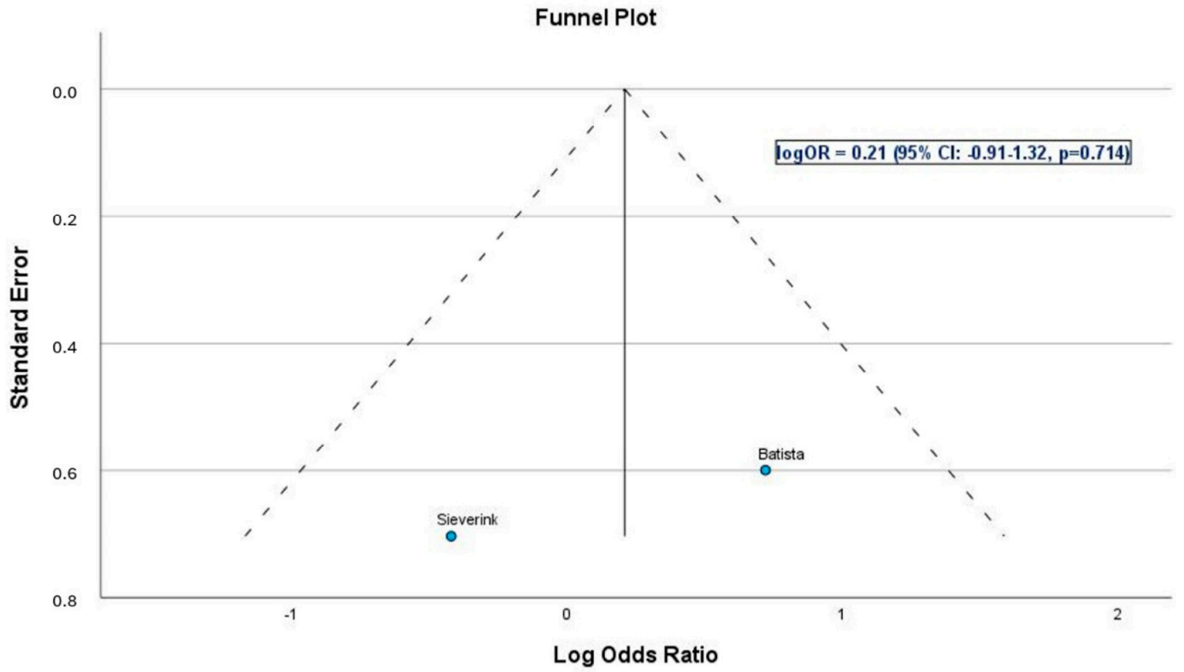

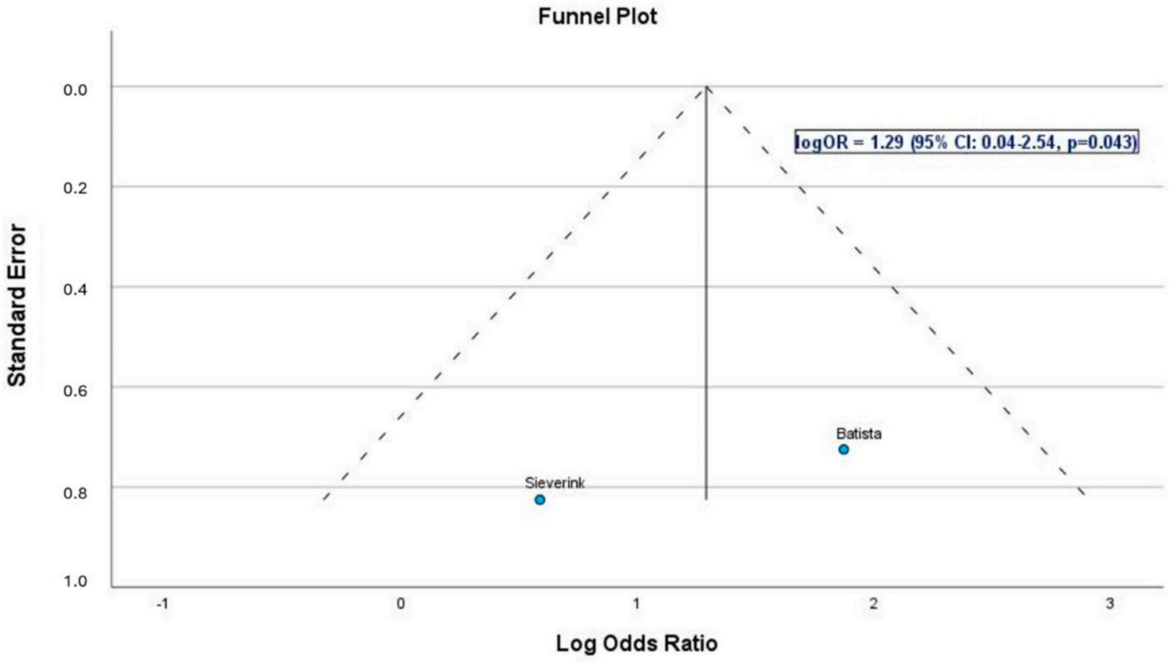
Funnel Plot
logOR = 1.29 (95% CI: 0.04-2.54, p=0.043)
Standard Error
0.0
0.2
0.4
0.6
0.8
1.0
Batista
Sieverink
-1
0
1
2
3
Log Odds Ratio

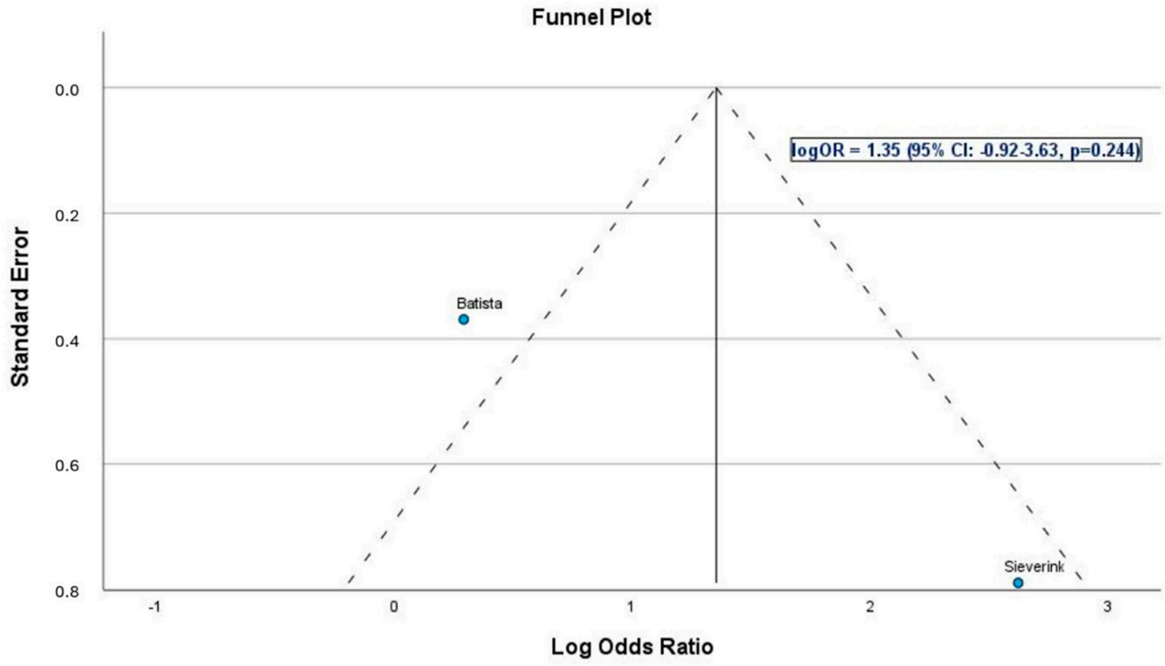
Funnel Plot
logOR = 1.35 (95% CI: -0.92-3.63, p=0.244)
Standard Error
0.0
0.2
0.4
0.6
0.8
Batista
Sieverink
-1
0
1
2
3
Log Odds Ratio

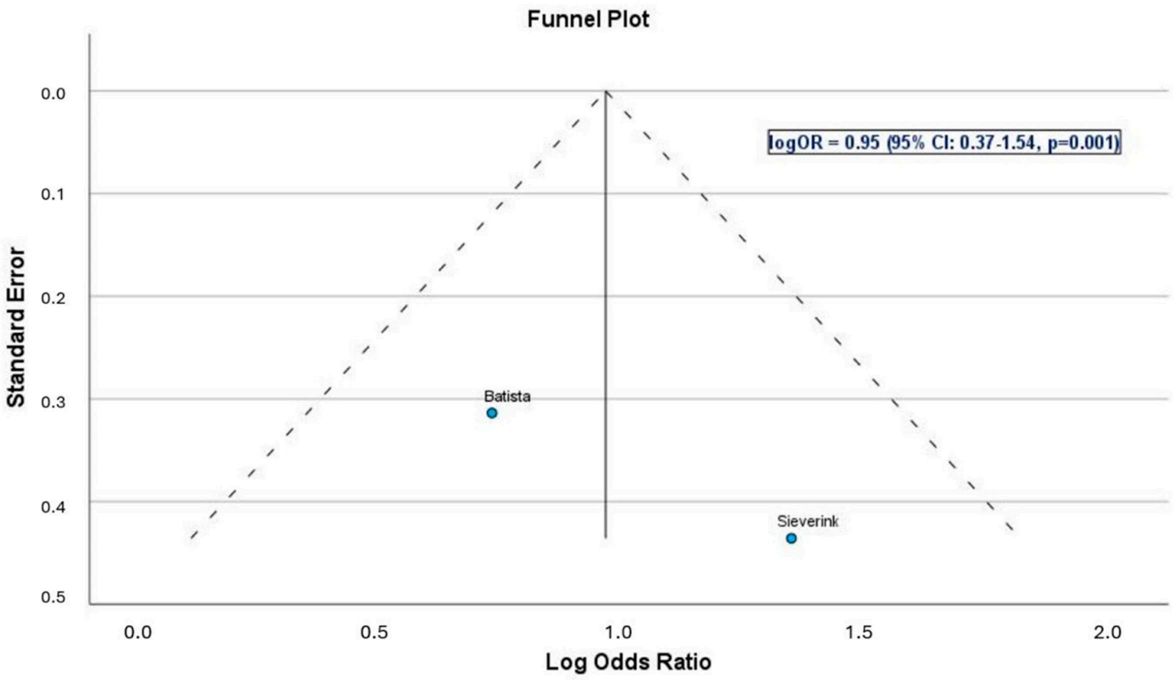

Legend: Supplement Figure S1a-e display Funnel Plots for the five endpoints: Sensitivity (1a), Specificity (1b), Positive Predictive Value (1c), Negative Predictive Value (1d), and Accuracy (1e).

Funnel Plots are used to visualize the precision of studies or analyses and identify potential biases due to publication bias or other factors. The X-axis represents the Log Odds Ratio, while the Y-axis represents the Standard Error. In these plots, a symmetrical Funnel Plot suggests low bias and sufficient precision, whereas asymmetry may indicate possible bias or heterogeneity.

Note: The Effect-Size, along with its corresponding 95% confidence interval, has been included within the Funnel Plots.

Figure S2a-e: Galbraith Plots

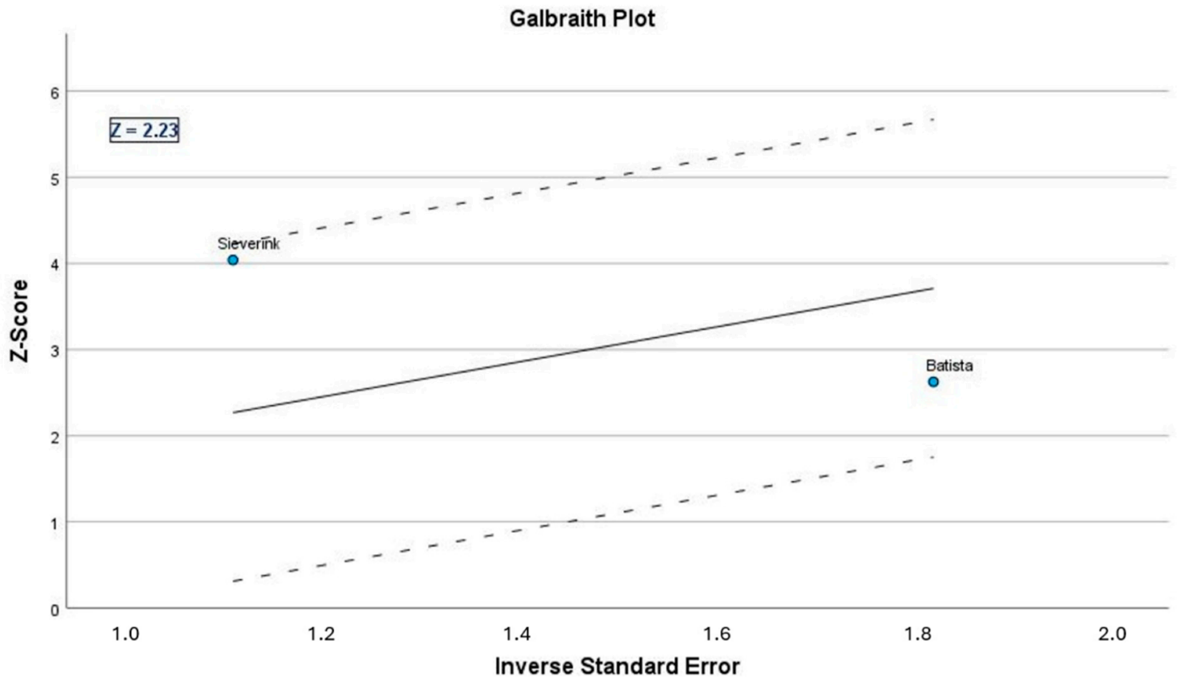

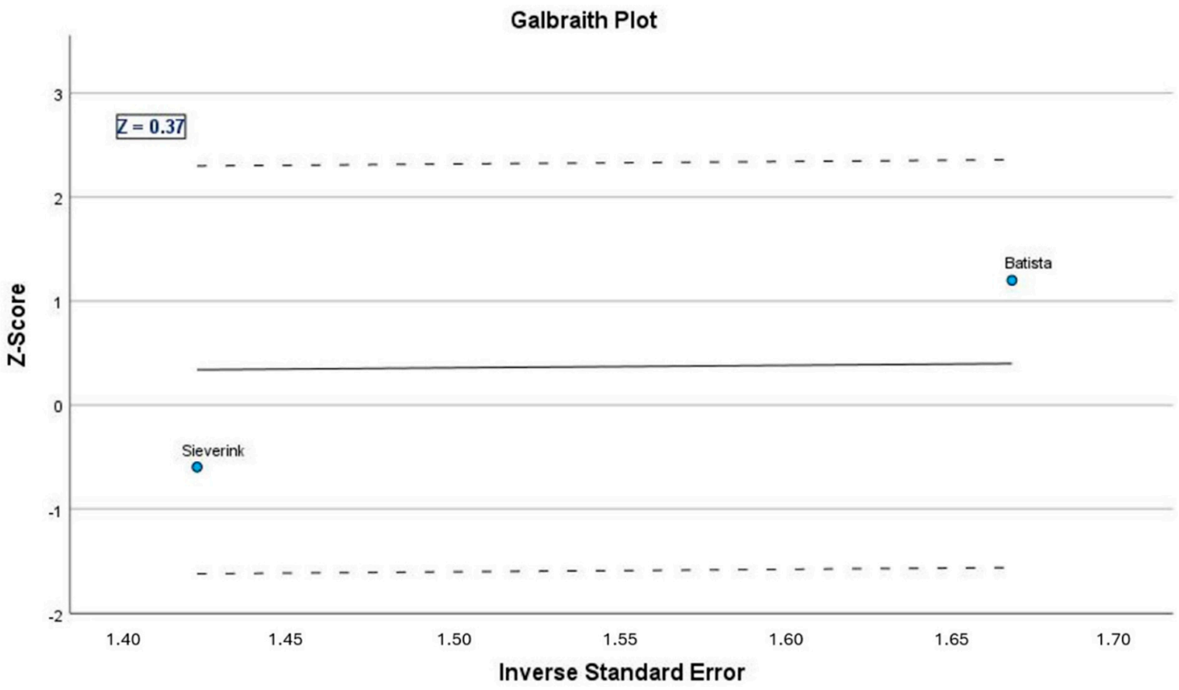

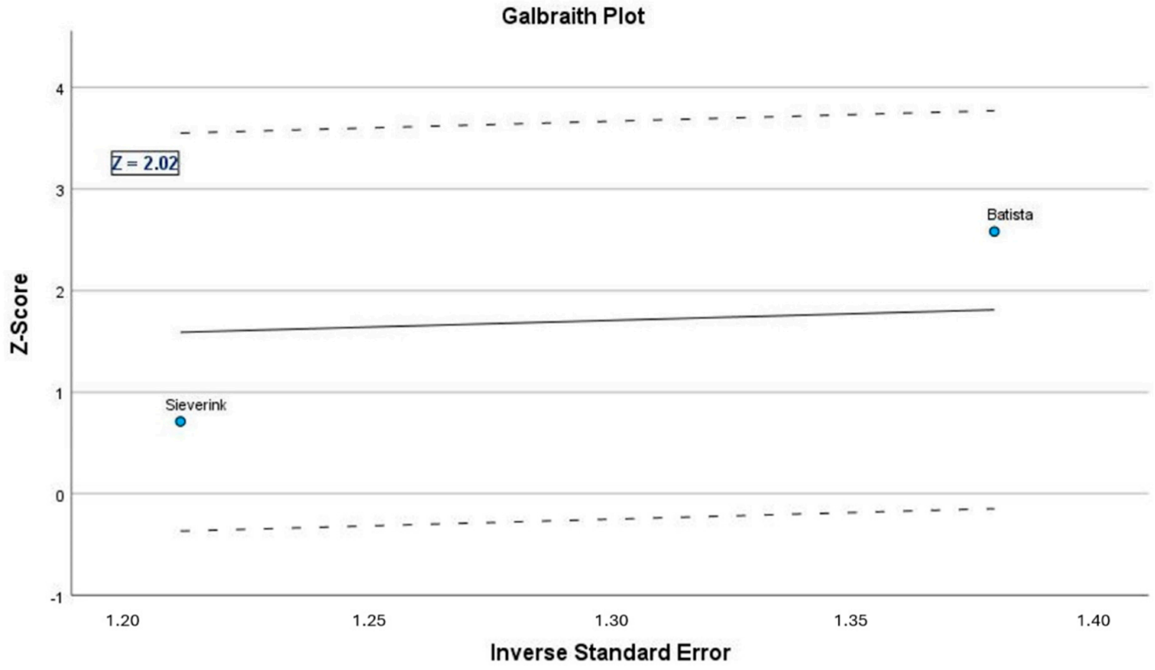
Galbraith Plot
Z = 2.02
Batista
Sieverink
Z-Score
4
3
2
1
0
-1
1.20
1.25
1.30
1.35
1.40
Inverse Standard Error

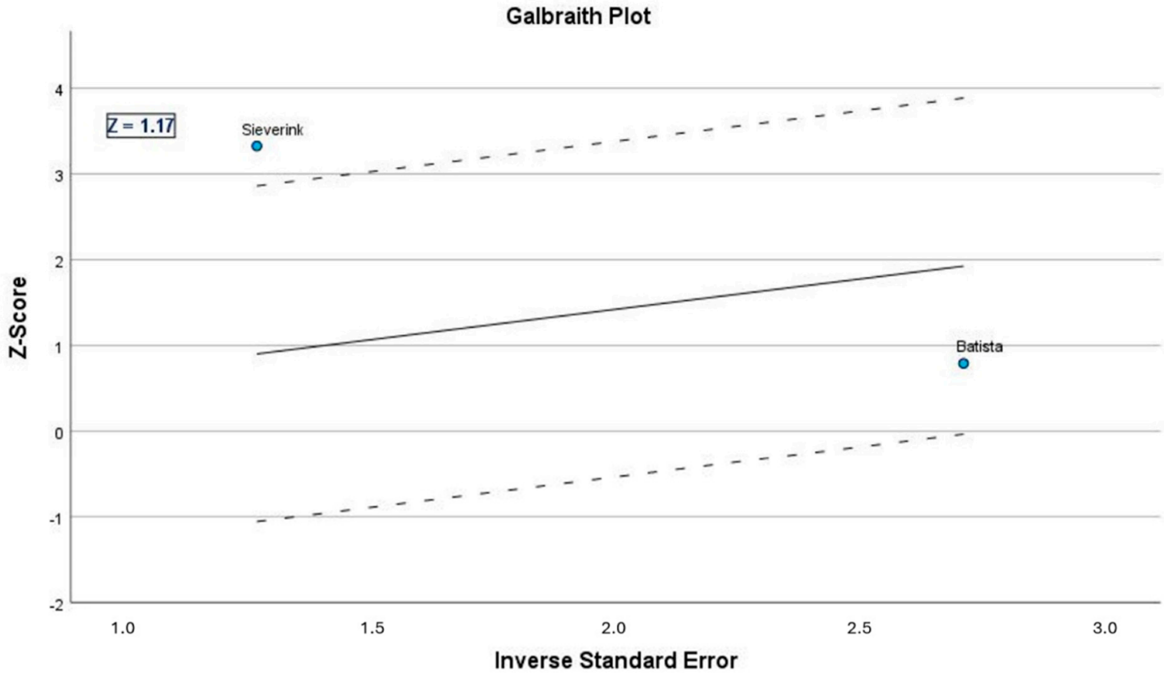
Galbraith Plot
Z = 1.17
Sieverink
Batista
Z-Score
4
3
2
1
0
-1
-2
1.0
1.5
2.0
2.5
3.0
Inverse Standard Error

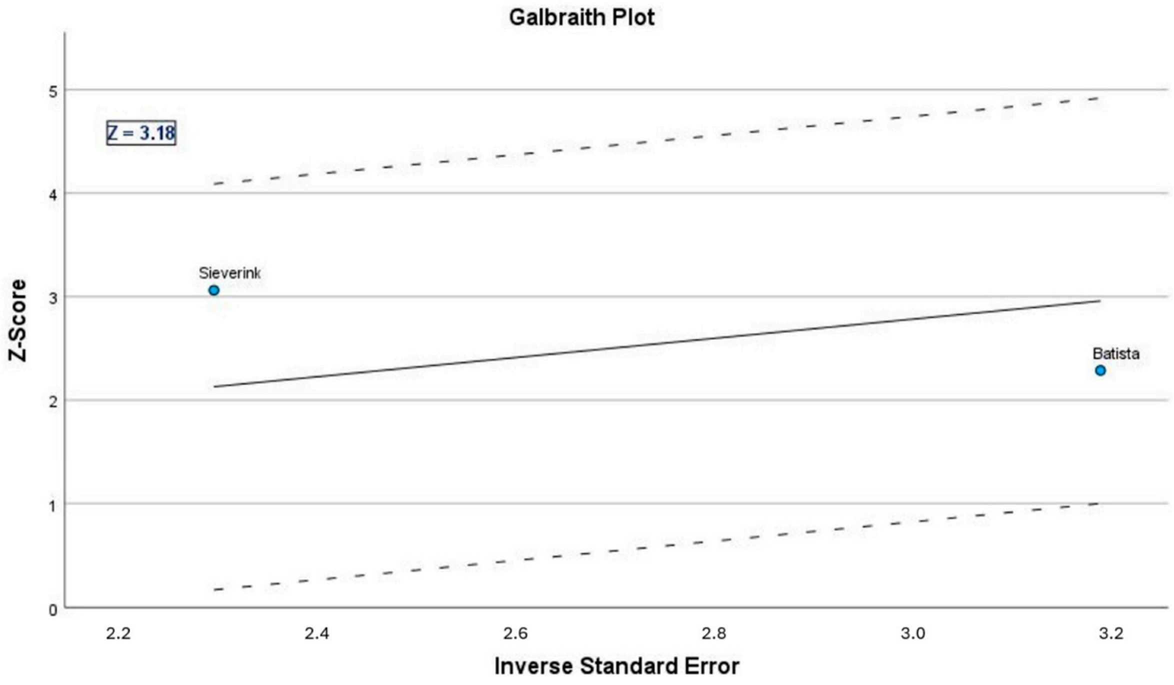

Legend: Supplement Figure S2a-e depict Galbraith Plots for the five endpoints: Sensitivity (2a), Specificity (2b), Positive Predictive Value (2c), Negative Predictive Value (2d), and Accuracy (2e). Galbraith Plots are used to investigate heterogeneity between studies or analyses. In these plots, the X-axis represents the inverse of the Standard Error, and the Y-axis represents the Z-Score. When points in a Galbraith Plot fall within the confidence intervals (estimation intervals) around the zero line, it indicates homogeneous results. Deviations from the zero line may suggest the presence of heterogeneity, necessitating further analysis to identify the sources of this variation.

Figure S3: Risk of Bias plot

Risk of Bias (RoB) assessment of four studies included (see references in the main manuscript [19,20,25,26]).

Low: low risk of bias introduced by study methods

High: high risk of bias introduced by study methods

Unclear: unclear risk of bias due to insufficient data reported to permit a judgment

RISK OF BIAS				
**Study**	**Patient Selection**	**Index Test**	**Reference Standard**	**Flow & Timing**
Sieverink, 2020	Low	Low	Unclear	Unclear
Batista, 2019	Unclear	Low	Unclear	Unclear
Azawi, 2023	Unclear	Low	Unclear	Unclear
Ramos, 2023	Low	Low	Low	Unclear

APPLICABILITY CONCERNS			
**Study**	**Patient Selection**	**Index Test**	**Reference Standard**
Sieverink, 2020	Low	Low	Unclear
Bastia, 2019	Unclear	Low	Unclear
Azawi, 2023	Unclear	Low	Unclear
Ramos, 2023	Low	Low	Low

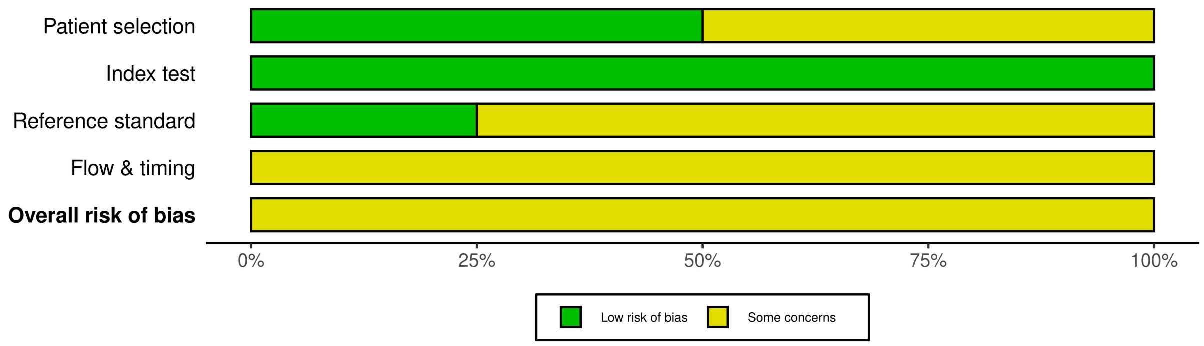

Legend: Supplement Figure S3 presents the Risk-of-Bias Assessment, considering the QUADAS criteria and the graphical representation according to McGuinness and Higgins.

see: Whiting PF, Rutjes AW, Westwood ME, et al. QUADAS-2: a revised tool for the quality assessment of diagnostic accuracy studies. Ann Intern Med. 2011 Oct 18;155(8):529-536. doi: 10.7326/0003-4819-155-8-201110180-00009. [23] *and:* McGuinness LA, Higgins JPT. Risk-of-bias VISualization (robvis): An R package and Shiny web app for visualizing risk-of-bias assessments. Res Synth Methods. 2021 Jan;12(1):55-61. doi: 10.1002/jrsm.1411. Epub 2020 May 6. [24].

Table S3: Certainty of Evidence (CoE) of the recommendation according to GRADE (for every of the five endpoints).

Study objectives	Number of results per 1000 patients tested	Number of tests (studies)	CoE (GRADE)
True positives	119	1190 (4)	⊗⊗∅∅ (low)
False negatives	30		
True negatives	825	1190 (4)	⊗⊗∅∅ (low)
False positives	26		
Inconclusive test results	Not reported (it is to be assumed from zero)		
Complications arising from the diagnostic test	Not reported (it is to be assumed from zero)		

CoE according to GRADE:

⊗⊗⊗⊗, high

⊗⊗⊗∅, moderate

⊗⊗∅∅, low

⊗∅∅∅, very low

<u>Question:</u> Should Uromonitor® test be used to diagnose (nearly 15% prevalence) urothelial carcinoma of the bladder (UCB) in patients?

(In order to assess the "Certainty of Evidence" (CoE) within one of the four categories (see above), the criteria of study design, accuracy of test results, and sample size are considered alongside the domains of risk of bias, imprecision, inconsistency, indirectness, and publication bias pertaining to the study objectives.)

see: Schunemann HJ, Oxman AD, Brozek J, et al. Grading quality of evidence and strength of recommendations for diagnostic tests and strategies. BMJ. 2008 May 17;336(7653):1106-1110. doi: 10.1136/bmj.39500.677199.AE. [22].

Figure S4: Workflow of the Uromonitor® test in patients under surveillance for non-muscle-invasive bladder cancer

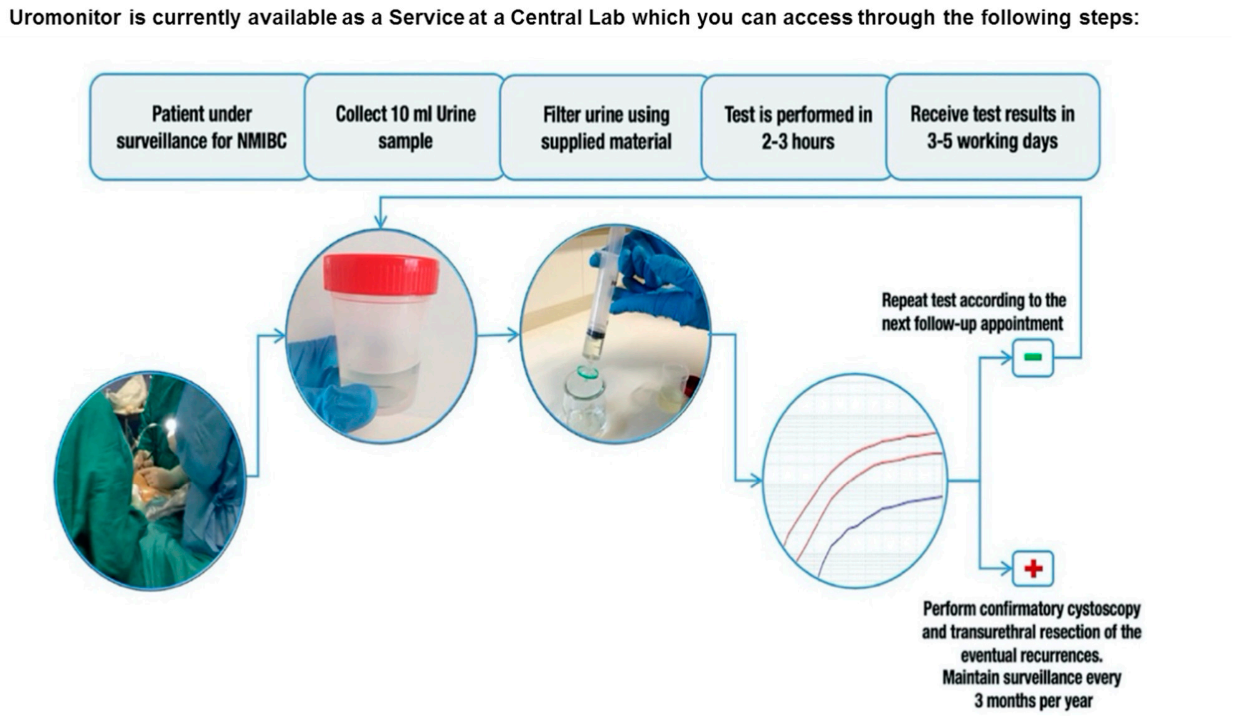

Legend: Urine testing workflow. In patients under surveillance for non-muscle-invasive bladder cancer (NMIBC), a minimum of 10 ml of urine is collected before cystoscopy. This 10 ml of urine is then filtered through a 0.8-µm filter and stored at 4°C. DNA extraction and Uromonitor® test are then performed. If a positive result is obtained, confirmatory cystoscopy and transurethral resection of eventual recurrences are recommended. If a negative result is obtained, it is recommended that the test should be repeated on next follow-up appointment.

Instructions or a guide on how to use the Uromonitor® test in the form of a video:

https://www.youtube.com/watch?v=UgnfL3-hH6Y
